# Pesticide exposure affects flight dynamics and reduces flight endurance in bumblebees

**DOI:** 10.1002/ece3.5143

**Published:** 2019-04-29

**Authors:** Daniel Kenna, Hazel Cooley, Ilaria Pretelli, Ana Ramos Rodrigues, Steve D. Gill, Richard J. Gill

**Affiliations:** ^1^ Department of Life Sciences Imperial College London Silwood Park Ascot Berkshire UK; ^2^ Dipartimento di Biologia Università di Padova Padova Italy; ^3^ Department of Human Behaviour, Ecology, and Culture Max Planck Institute for Evolutionary Anthropology Leipzig Germany

**Keywords:** *Bombus terrestris audax*, flight mill, foraging, Imidacloprid, neonicotinoid, velocity

## Abstract

The emergence of agricultural land use change creates a number of challenges that insect pollinators, such as eusocial bees, must overcome. Resultant fragmentation and loss of suitable foraging habitats, combined with pesticide exposure, may increase demands on foraging, specifically the ability to collect or reach sufficient resources under such stress. Understanding effects that pesticides have on flight performance is therefore vital if we are to assess colony success in these changing landscapes. Neonicotinoids are one of the most widely used classes of pesticide across the globe, and exposure to bees has been associated with reduced foraging efficiency and homing ability. One explanation for these effects could be that elements of flight are being affected, but apart from a couple of studies on the honeybee (*Apis mellifera*), this has scarcely been tested. Here, we used flight mills to investigate how exposure to a field realistic (10 ppb) acute dose of imidacloprid affected flight performance of a wild insect pollinator—the bumblebee, *Bombus terrestris audax*. Intriguingly, observations showed exposed workers flew at a significantly higher velocity over the first ¾ km of flight. This apparent hyperactivity, however, may have a cost because exposed workers showed reduced flight distance and duration to around a third of what control workers were capable of achieving. Given that bumblebees are central place foragers, impairment to flight endurance could translate to a decline in potential forage area, decreasing the abundance, diversity, and nutritional quality of available food, while potentially diminishing pollination service capabilities.

## 1 INTRODUCTION

The extent to which insects move across landscapes has significant implications for human welfare. Highly mobile species can potentially cause detrimental insect pest outbreaks (Mazzi & Dorn, 2012; Sharov & Liebhold, 1998), invasions (Myers, Simberloff, Kuris, & Carey, 2000; Renault, Laparie, McCauley, & Bonte, 2018), or the spread of vector‐borne diseases (Dujardin et al., 2008; Estrada‐Peña, Ostfeld, Peterson, Poulin, & de la Fuente, 2014; Githeko, 2000; Rogers & Packer, 1993), yet, insect movement can also underpin beneficial ecosystem service provision. For example, the majority of angiosperms, including around ¾ of our crop species, are to some degree reliant upon the extensive movement of foraging insect pollinators (Gill et al., 2016; Kleijn et al., 2015; Klein et al., 2007; Ollerton, Winfree, & Tarrant, 2011). It is therefore important we understand which, and to what extent, stressors affect insect pollinator flight performance if we are to mitigate threats to a global pollination service valued at >€150 bn annually (Benaets et al., 2017; Fischer et al., 2014; Gallai, Salles, Settele, & Vaissière, 2009; Gill & Raine, 2014; Stanley et al., 2015; Wolf et al., 2014).

The emergence of intensive agriculture can cause loss and fragmentation of suitable foraging habitats, leading to resources becoming increasingly sparse and isolated within an insect's foraging range (Didham, Ghazoul, Stork, & Davis, 1996; Hadley & Betts, 2012; Steffan‐Dewenter & Tscharntke, 1999; Tscharntke & Brandl, 2004; Zurbuchen et al., 2010). This may pose a considerable challenge for eusocial bees, which are central place foragers having a fixed nest site. Workers must undertake return foraging trips from this set nest location, and consequently any habitat discontinuity may require workers to fly longer distances to find and bring back resources, such as pollen and nectar (Goulson, Lye, & Darvill, 2008; Jha & Kremen, 2013; Pelletier & McNeil, 2003; Schmid‐Hempel & Schmid‐Hempel, 1998). Hence any stressor lowering individual worker flight ability could translate to negative colony level impacts (Gill, Ramos‐Rodriguez, & Raine, 2012), with implications for the crucial ecosystem services they provide (Delaplane & Mayer, 2000; Garibaldi et al., 2013; Greenleaf & Kremen, 2006; Potts et al., 2010; Winfree, Williams, Gaines, Ascher, & Kremen, 2008).

Insecticides are commonly applied in agricultural landscapes as a pest management strategy (Fernandez‐Cornejo & Vialou, 2014; Ramankutty et al., 2018), with neonicotinoids being one of the most widely used classes worldwide (Simon‐Delso et al., 2015). However, neonicotinoids have been implicated as a threat to eusocial bees (Gill et al., 2012; Goulson, 2013; Lundin, Rundlöf, Smith, Fries, & Bommarco, 2015; Tsvetkov et al., 2017; Whitehorn, O'Connor, Wackers, & Goulson, 2012; Woodcock et al., 2017). Foraging eusocial bees are frequently exposed to neonicotinoids in treated landscapes (Botías, David, Hill, & Goulson, 2017; Botías et al., 2015; David et al., 2016; Mitchell et al., 2017; Rolke, Persigehl, Peters, Sterk, & Blenau, 2016), and controlled exposure experiments have demonstrated impaired homing ability (Fischer et al., 2014) and foraging efficiency of workers, including longer foraging trips and reduced rate of pollen collection (Feltham, Park, & Goulson, 2014; Gill & Raine, 2014; Stanley & Raine, 2016). A possible explanation for these reported impairments is that certain aspects of foraging flight dynamics, such as endurance and speed, are affected by neonicotinoid exposure. However, to date only two studies (both using tethered honeybees) have specifically tested this and have reported mixed findings. One study found acute neonicotinoid (118 ppb thiamethoxam) exposure increased flight endurance, with the opposite effect shown following chronic (32.5–45 ppb thiamethoxam) exposure (Tosi, Burgio, & Nieh, 2017). The other study detected a negative effect of chronic (6 ppb imidacloprid) exposure on flight distance, but only when provided to individuals in combination with the parasitic varroa mite (Blanken, van Langevelde, & van Dooremalen, 2015). Hence, further investigation is needed to understand the generality of the effects of exposure on bee flight, while also: (a) ensuring that a concentration within the field realistic range is used; (b) gaining a more in‐depth analysis of the dynamics of flight during testing; (c) investigating a representative species of wild bee, given there can be differential responses to pesticide exposure among insect pollinator species (Cresswell et al., 2012; Heard et al., 2017; Rundlöf et al., 2015); and (d) considering variation in worker body size, given this can modulate flight capability, can be associated with variation in foraging behaviors within bumblebee colonies (Goulson et al., 2002; Spaethe & Weidenmuller, 2002), and size‐specific energetic demands show a nonlinear positive relationship (Greenleaf, Williams, Winfree, & Kremen, 2007; Kaufmann, Reim, & Blanckenhorn, 2013).

**Figure 1:**
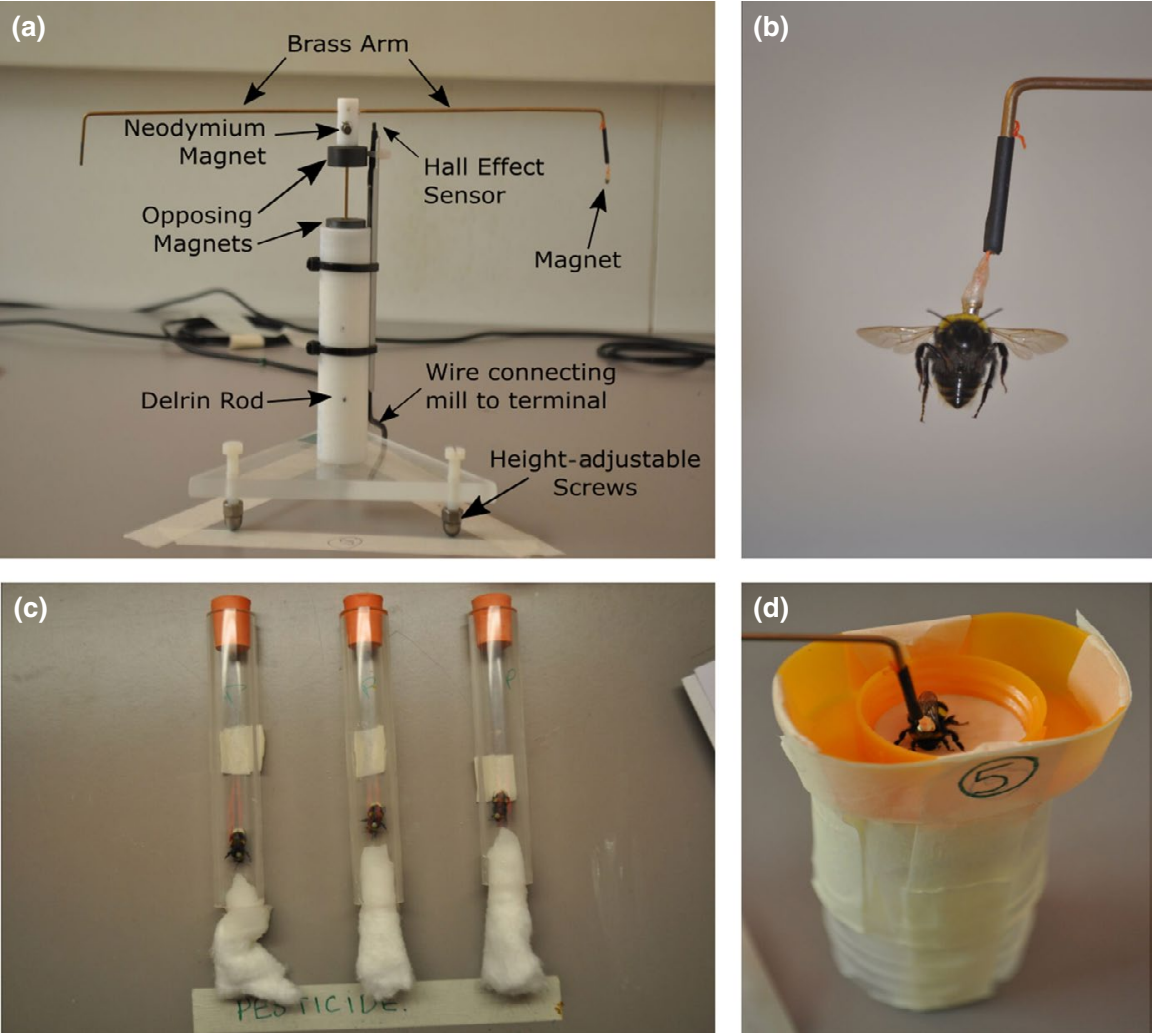
Flight mill setup and associated experimental procedures. Panels show (a) flight mill used in the study (the “height‐adjustable screws” ensured the mill could be horizontal with an attachable bubble level used to ensure this); (b) tethering of an individual worker bumblebee to the flight mill magnet; (c) feeding procedure in which workers were placed in bunged tubes with one end consisting of cotton wool lightly soaked in 50% sucrose solution (with or without 10 ppb imidacloprid); (d) support stands used to hold workers prior to flight tests and following a stop in flight

We investigated the effects of acute oral neonicotinoid exposure on different aspects of bumblebee (*Bombus terrestris audax*) flight performance using a controlled tethered flight mill setup (Figure 1). For this study, we exposed individual workers to the neonicotinoid imidacloprid at a concentration of 10 ppb as it is: (a) a widely used insecticide across the globe with a growing market in many regions (Auteri et al., 2017; Casida, 2018; Cressey, 2017; Mitchell et al., 2017; Zhang, 2018); (b) a concentration that can be found inside social bee colonies, on return foraging workers, and in the pollen and nectar of individual flowers (Blacquière, Smagghe, van Gestel, & Mommaerts, 2012; Botías, David, Hill, & Goulson, 2016; Cresswell, 2011; Dively & Kamel, 2012; Goulson, 2013; Hladik, Vandever, & Smalling, 2016); (c) known to impair foraging performance after exposure (Godfray et al., 2014; Pisa et al., 2017); and (d) a neonicotinoid under current scrutiny by policy makers and regulators (Cressey, 2017), resulting in a recent EU ban from agricultural use outside of closed greenhouses. Here, we tested the propensity of individual bees to fly, followed by the measures of their flight distance and duration, the dynamics of velocity over the course of the flight test, and investigated how neonicotinoid exposure interacted with worker body size on these performance measures.

## 2 METHODS

### 2.1 Bee husbandry

Three bumblebee *Bombus terrestris audax* colonies, containing a queen and between 130 and 150 workers, were supplied by a commercial company (Agralan Ltd). Each colony was delivered in a separately housed plastic nest box (29 × 22.5 × 13 cm) and kept in a controlled environment room (25°C) under red light. From the point of arrival, colonies were provisioned with 4g of pollen daily and supplied with ad libitum 10/90% sucrose/water solution via a connected reservoir. A 10% sucrose concentration falls within the range of many flower species (Pierre, Mesquida, Marilleau, Pham‐Delegue, & Renard, 1999; Pyke & Waser, 1981) and was sufficient to ensure that provisioned colonies did not suffer from dehydration or starvation. However, our primary justification for using this relatively low concentration in the nest was to increase the motivation of individual workers to feed when provided with a higher concentration of sucrose solution during the acute exposure setup. This was necessary to facilitate sufficient uptake of pesticide active ingredient in exposed workers and increased the likelihood that all workers (control or treatment) would feed to satiation prior to flight.

### 2.2 Flight mill setup and bee tethering

Six flight mills were set up in a separate adjoining room under the same environmental conditions as the housing room (constant 25°C temperature), but with the option to switch between red (Philips TLD 58W Red 1SL/25; mean 660 λ nm) and white light (Philips TLD 58W 840). Flight mills were adapted from a previous design (Smith & Jones, 2012), consisting of a revolving brass wire, with a magnet hanging from one end designed to attach to a metal tag glued to the bee's thorax through magnetic attraction (Figure 1). The revolving brass wire was suspended over a central Delrin rod by the repulsive forces of two magnets, preventing friction during arm rotation to allow fluid motion. The Delrin rod was positioned vertically (90° perpendicular) on a horizontally flat triangular Perspex base. A digital Hall effect sensor placed on one side of the mill detected each complete revolution by the passage of a neodymium magnet (Figure 1a) and sent an impulse to a Raspberry Pi computer (model B) via a copper wire connector. From here, a Python script recorded the time (in seconds) between each impulse, with each revolution defined as a “circuit” from hereon.

**Figure 2:**
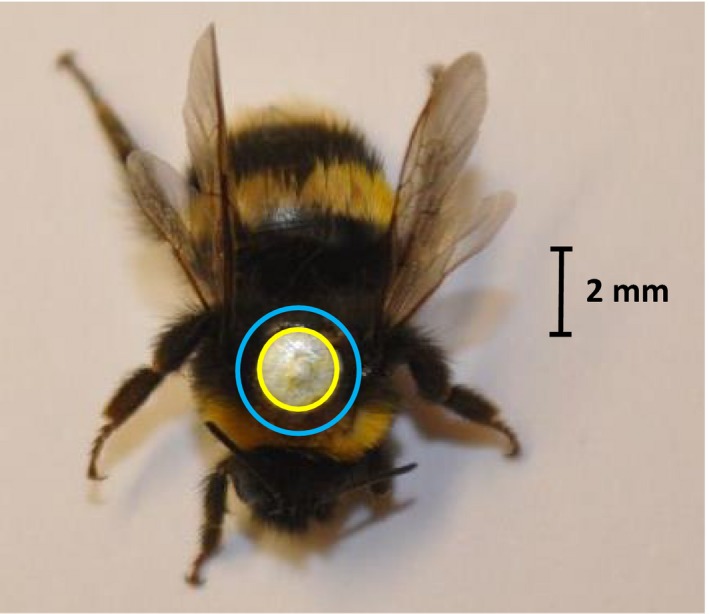
Example of the ideal positioning of a metal tag (tag score 1) on the thorax of a *Bombus terrestris audax* bumblebee worker. If the tag positioning was unideal (tag score 2), the metal tag would overlap the yellow circle but remains inside the blue circle. If positioning was unacceptable (tag score 3), it would overlap the blue circle

When the colonies arrived, we randomly selected 110 workers per colony (total = 330) and under red light attached a circular galvanized iron tag (diameter = 2 mm, thickness = 0.4 mm) to the thorax of each worker using super glue (Figure 2), allowing each individual to be tethered to the hanging flight mill magnet (Figure 1b, d). We were confident that tag mass would not cause any significant impairment to bee flight performance, as mean (±*SEM*) tag mass was 18 ± 0.3 mg (calculated from weighing 30 tags), equating to just 7.5% of the mean worker wet mass of all individuals tested in this study (240 ± 5 mg). Indeed, bumblebees are capable of carrying >50% of their own body mass in nectar alone when foraging (Brian, 1954). Each tag was placed at the center of the thorax, with the tag leading edge touching the back of the first thoracic stripe (Figure 2). This placement ensured no impediment of wing movement when attached to the flight mill. The meticulous nature of tagging each bumblebee meant that we scored tag positions as 1 = ideal, 2 = unideal, or 3 = unacceptable (Figure 2), with scores 1 and 2 being considered acceptable to experimentally test but score 3 being excluded from further use. In total, 74 workers per colony were experimentally tested (total = 222).

### 2.3 Pesticide preparation

A 10 ppb imidacloprid working solution was produced to supply the acute pesticide exposure to worker individuals. A previously made stock solution of imidacloprid dissolved in acetone at a 1 ppt concentration was used, which was stored in a freezer wrapped in aluminum foil to prevent light degradation (Soliman, 2012). Aliquots of the stock solution were diluted by the addition of a 50% sucrose solution to create the working 10 ppb *pesticide* treatment solution. The control solution was made by adding the respective volume of acetone to 50% sucrose solution to produce a 10 ppm acetone *control* working solution.

### 2.4 Experimental procedure

Pilot studies were first conducted in September 2015 and March 2017 to trial and verify the experimental setup and procedures, with the main experimental study conducted in April 2017. The main experimental testing started 12 hr after tagging was completed, and testing took place over an 8‐day period. Workers were tested in bouts, with 5–6 bouts undertaken per day. Six workers were sampled per bout (one per flight mill), consisting of two workers sampled per colony, with one worker randomly assigned to the *treatment* and the other to the *control*. This ensured that all three colonies and both treatment groups were represented equally in each bout and over the totality of the experiment (total: *n* = 37 bees per treatment per colony; *n* = 111 *control* & 111 *pesticide*). Once removed from the colony, each worker was directly transferred to a separate transparent horizontally laid Perspex tube (length = 150 mm, internal diameter = 19 mm). The tube had a rubber bung at each end creating a holding compartment for the bee, with individuals left to acclimatize for a resting period of 3 min. After this resting period, one of the bungs was replaced with cotton wool lightly soaked in the *control* or *pesticide* treatment sucrose solution (Figure 1c). This already piloted method ensured that 94.1% of workers in our main experiment fed. We made the assumption that spiking the sucrose solution with the neonicotinoid would not deter feeding if given no other option, as supported by pilot observations and previous studies (Arce et al., 2017; Gill et al., 2012; Kessler et al., 2015). Our pilot study also indicated that workers took a mean (±*SEM*) duration of 50 ± 13 s to commence feeding, defined as prolonged (>2 s) proboscis extension on to the cotton wool, and fed for a mean (±*SEM*) duration of 213 ± 24 s before stopping, with subsequent feeds being rare, sporadic, and short (<10 s). Workers could access the provisioned sucrose‐soaked cotton wool for 10 min, after which the cotton wool was removed and original bung replaced, followed by a 5‐min resting period inside the tube. While this protocol meant that we could not determine the precise dosage of imidacloprid consumed by each worker, which might have improved the predictive power of our models, it did allow workers to feed to satiation, which is a state likely to occur in the field during foraging bouts and allowed consumption volume to vary proportionately to individual worker size (Free & Butler, 1959; Goulson et al., 2002).

The workers that fed were then removed carefully using tweezers and tethered to the flight mill. The 5.9% of workers that did not feed were immediately frozen (−20°C) and weighed along with all other bees after all flight tests had been completed. All of this was carried out under red light conditions, but once workers were tethered to the mills, the room was switched to white light. Each mill had a separate height‐adjustable stand which was erected once the bee was tethered and used to hold the worker in place (Figure 1d). Prior to initiating the flight test, workers were held in place for a period of 10 min for two primary reasons. Firstly, pilot observations demonstrated that some bees were initially irritated by attachment to the mill and would attempt to dislodge themselves from the magnet using middle and hind legs, which discouraged flight. A 10‐min acclimatization period allowed irritation to subside, and all bees in the pilot study had withdrawn legs from the magnet at this point. Secondly, a balance was sought between giving workers time to metabolize the neonicotinoid and preventing demotivation to fly by having them separated from their natal colony for too long. Honeybees metabolize imidacloprid and other neonicotinoids quickly, with a 100 µg/kg dose of imidacloprid showing the greatest levels of presence in the thorax and abdomen after just 20 min from ingestion (Suchail, De Sousa, Rahmani, & Belzunces, 2004), and >50% of a 100 µg/kg dose of acetamiprid being metabolized in less than 30 min (Brunet, Badiou, & Belzunces, 2005). In our study, a total of 25 min passed from starting the feeding trial to starting the flight test, which we are therefore confident, represent enough time for absorption and metabolism of some of the imidacloprid consumed.

Immediately after the 10‐min acclimatization period, the support stand was removed quickly from beneath the bee to stimulate flight. Prior to removal, the stand was rotated to ensure the worker had a forward‐facing orientation. Stand removal caused the loss of tarsal contact with the stand surface, which can trigger flight as evidenced in our pilot and other previous studies (Blanken et al., 2015; Brodschneider, Riessberger‐Gallé, & Crailsheim, 2009; Tosi et al., 2017). However, if the worker did not initially start flying, the flat side of the stand was used to gently tap the legs in order to generate a sharp loss of tarsal contact. Up to three taps were allowed in this first flight attempt, with individuals being removed from the flight test if no flight was initiated.

Workers that successfully flew in the first attempt were monitored for any subsequent flight stoppages. Each flight stoppage was noted, and each worker was permitted five stoppages before their flight test was terminated. Immediately following a flight stoppage, the individual would be held in the stand to ensure tarsal contact for a 20 s rest period before removal of the stand again. Therefore, in the subsequent data analysis, flight stoppages were identified as circuits with a duration >20 s. After a stoppage, workers were only permitted one tap of the legs in an attempt to trigger flight; otherwise, their flight test was terminated. Any stoppages that occurred on the first circuit were discounted as genuine stoppages, as this was deemed an acclimatization circuit for bees to familiarize themselves with the experimental setup. All workers were given the opportunity to fly for up to 60 min, including all stops, after which the flight test was terminated. Each individual worker was allowed a maximum of five flight stoppages, essentially allowing each bee five chances to continue its flight until the 60 min end point. This provided a better representation of field conditions and a more realistic prediction of foraging distances, as foraging bees do not fly continuously during foraging bouts but will stop at flowers periodically to feed and rest (Woodgate, Makinson, Lim, Reynolds, & Chittka, 2016). Additionally, it allows individuals to acclimatize to the conditions of the flight mill and decreases the possibility of excluding individuals from testing that are initially demotivated to fly due to the experimental setup.

Following each flight test, workers were placed in separate labeled tubes and frozen (−20°C). After completion of the whole experiment, for each individual worker we measured: (a) wet body mass (including the attached metal tag; accuracy 0.1 mg); and (b) intertegula span (ITS; accuracy 0.01 mm) taken using a digital calliper (Workzone 150 mm), with the mean of three repeated measurements being used. For our data analysis, ITS was taken as a proxy for worker body size (Cane, 1987; Greenleaf et al., 2007). This is more appropriate than considering worker wet mass, as wet mass will vary according to both the volume of sucrose solution consumed and the duration of flight, as individuals gain mass through feeding and lose it through energy metabolism during flight.

### 2.5 Data cleaning

Frequency distribution plots revealed a spike in the number of workers that terminated the flight test before completing 100 m (118 circuits; Supporting information Figure S1a). Workers that did not fly over this threshold distance were excluded from the endurance and velocity analysis as a precautionary measure to discount individuals whose flight mill performance is not representative of actual flight capacity. For each worker flying beyond the 100‐m threshold, we calculated the following: (a) total distance flown during the flight test, by taking the total number of circuits flown multiplied by the circuit circumference (0.848 m); (b) total duration of the flight test, by summing all circuit interval times; and (c) velocity of each circuit, by taking the circuit circumference and dividing it by the respective circuit interval time. We took a simple calculation for mean velocity, calculated as the total distance flown divided by the total duration flown, and maximum velocity was taken from the circuit showing the highest velocity attained across the flight test.

The velocity calculations for each individual flight test were carried out on cleaned data in which the following circuits were excluded from the analysis: (a) first five circuits of the first flight attempt; (b) first five circuits directly following a flight stoppage; (c) the circuit directly preceding a flight stoppage. It was noted from pilot observations and the main study that removal of the support stand or tapping of the legs would often stimulate strikingly high velocities. It is likely this behavior is a reaction to stimulatory stress, so we felt actions (a) and (b) were justified as a precautionary measure to ensure we only considered circuits representative of normal continuous flight. Similarly, in justification of action (c), the minimal rotational resistance of the mill means that when a worker stops flying it does not equate to an abrupt stop, but the brass arm continues to rotate and slows gradually.

### 2.6 Data and statistical analysis

When considering total duration flown, it was noted that the data were bimodally distributed (Supporting information Figure S1b); therefore, we converted the results to a binary response variable categorized as having or having not flown >2000 s, with this duration value decided on as it fell at the bottom of the bimodal concave.

Statistical analyses were conducted using the “lme4” (Bates, 2015) package in R v3.2.0 (R Core Team, 2015), with summary statistics generated using the package “psych” (Revelle, 2015) and results reported using the package “lmerTest” (Kuznetsova, Brockhoff, & Christensen, 2015). A linear model was used to compare variation in *ITS* (body size) between treatments, with *treatment* (*control* or *pesticide*) as the only fixed effect. For all other analyses, mixed‐effects models (fitted by maximum likelihood) were initially used with *colony* included as a random factor. Unless otherwise stated, fixed effects in each analysis included *treatment* (*control* or *pesticide*), *ITS,* and the associated interaction term. Where response variables were binary (propensity to feed, propensity to fly, flight over 100 m, flight longer than 2000 s), the data were analyzed using a generalized linear mixed model (GLMM) function under a binomial family distribution, with a linear mixed model (LMM) function used for all other responses (feeding time, total distance flown, mean velocity, maximum velocity). However, where the random effect of *colony* explained none of the variance in the data, it was removed from the model, and the model reverted to either a generalized linear model (GLM) or linear model (LM), with the type of model used indicated with each result. To examine whether an unideal (score 2) tag fitting inhibited flight or impeded movement, we compared the propensity to fly (GLM) and distance flown (LM) between tag ratings for both *treatment* groups separately. Here, the fixed effects were *tag rating* (score of 1 = ideal or 2 = unideal), *ITS,* and the interaction between the two. Flight velocity over time (considering flight over the first 900 circuits) was analyzed using an LMM function with the random effect structure nesting individual bee ID within circuit to account for individual repeated measures over time and fixed effects including *treatment*, *ITS*, *circuit*, and the interaction term between *treatment* and *circuit*. The model suffered from high eigenvalues and had trouble converging when considering all 900 repeated measures; therefore to enhance model fit and convergence, we scaled the *circuit* variable and considered the average velocity of every 50th circuit (i.e., each bee had a mean per circuit velocity for circuits 1 to 50, 51 to 100, 101 to 150, and so on) resulting in 18 repeated measures. In all cases, model residuals were plotted to confirm the data met the parametric assumptions of the tests used. Where appropriate, normality tests were used to reveal distributions of the data, and those which appeared non‐normal were suitably transformed, with details of these found in Appendix S1. For all results, sample sizes are stated where appropriate, and as part of the statistical outputs, we provide in subscript the residual degrees of freedom from the respective model.

## 3 RESULTS

### 3.1 Feeding behavior

We found no significant effect of treatment on the propensity to feed (*n* = 9 *control* & 4 *pesticide* workers did not feed; GLM: *z*_219_ = 1.3, *p* = 0.20). In concordance with our pilot observations, we found that any feeds following the first were sporadic and short, suggesting workers fed to relative satiety on their first feed. Therefore, we used the length of first feeding time as a reliable proxy for total feeding time. Of the 209 workers that fed, the mean (±*SEM*) time spent feeding was 138 ± 9.0 s (*n* = 102) and 127.2 ± 7.8 s (*n* = 107) for *control* and *pesticide* workers, respectively, with no significant difference between treatments (LMM: *t*_204_=−0.8, *p* = 0.44; Supporting information Figure S2). We found that while body size was not a significant predictor of feeding time (LMM: *t*_204_ = 1.4, *p* = 0.17), the propensity to feed increased with increasing body size (GLM: *z*_219_ = 2.6, *p* = 0.008). For the main flight analysis, we decided to include only those workers that fed for >60 s (*control* = 86, *pesticide* = 94, total = 180; Table 1), because we wanted to increase the likelihood that each worker had fed to satiation. Additional analyses were run including all bees that fed (regardless of feeding time), which showed the same pattern of results to the main flight analyses detailed below, and these are reported in Supporting information Table S1.

**Table 1:** An overview of the filter steps used when cleaning the data for analysis of flight performance, outlining the number of workers removed from each treatment at each stage

	Control	Pesticide	Total
Total bees at start	**111**	**111**	**222**
Filter step 1			
Did not feed	9	4	13
Fed	**102**	**107**	**209**
Filter step 2			
Fed <60 s	16	13	29
Fed >60 s	**86**	**94**	**180**
Filter step 3			
Technical difficulties	2	2	4
Used in flight mill study	**84**	**92**	**176**
Filter step 4			
Tag Rating 2	18	18	36
Tag Rating 1	**66**	**74**	**140**
Filter step 5			
Did not fly	19	18	37
Flew	**47**	**56**	**103**
Filter step 6			
Flew <100 m	12	24	36
Flew >100 m	**35**	**32**	**67**
Filter step 7			
Removed top and bottom 10% sized individuals	9	5	14
Remaining bees for final analysis	**26**	**27**	**53**

### 3.2 Flight behavior

The flight data from 140 of the 180 bees tested on the flight mill were analyzed (Table 1), as four workers were not considered due to flight mill technical difficulties, and 36 not considered because unideal (score 2) tag application appeared to affect the aspects of flight performance (please see below for justification).

#### 3.2.1 Effect of tag fitting

The propensity of workers to fly was not significantly affected by tag rating (GLM: *control*; *z*_81_ = −1.0, *p* = 0.33: *pesticide*; *z*_89_ = −0.04, *p* = 0.97), although it is interesting that a higher percentage of tag rating 1 (ideal fitting) workers flew compared with tag rating 2 (unideal fitting) workers in both the *control* (71% vs. 61%) and *pesticide* (76% vs. 72%) groups. When considering total distance flown, however, tag rating 2 workers flew a significantly shorter mean distance compared with tag rating 1 bees in the *control* group (640 vs. 1,436 m, respectively; LM: *t*_55_ = −2.2, *p* = 0.03), with a similar, although nonsignificant, trend observed in the *pesticide* group (191 vs. 415 m; LM: *t*_66_ = −1.6, *p* = 0.11). Furthermore, we saw similar patterns in other flight metrics with tag rating 2 bees showing lower total duration flown (*control* = 1,114 vs. 2,132 s; *pesticide* = 272 vs. 553 s) and slower mean velocity (*control* = 0.562 vs. 0.657 m/s; *pesticide* = 0.618 vs. 0.744 m/s). It was therefore decided to exclude all 36 tag‐rated 2 workers (*control* = 18, *pesticide* = 18; Table 1) from our analyses, to avoid potential artefactual results.

#### 3.2.2 Initial flight behavior

**Figure 3:**
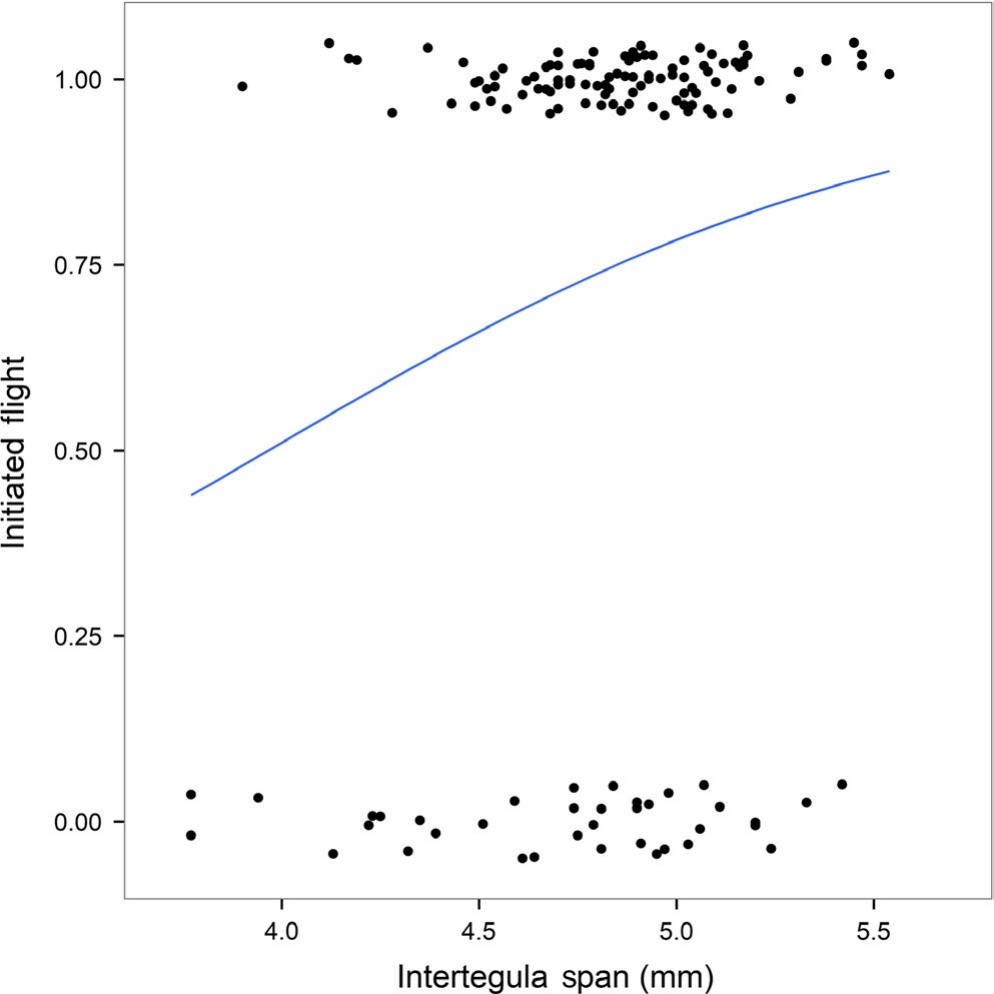
Logistic regression plot showing the effect of body size (*intertegula span*) on the propensity to fly. All workers from both treatments were pooled (*n* = 140) and could either have initiated flight (=1) or refused to fly (=0; Table 1 – filter step 4). Jitter has been added in the y‐plane so that individual data points are clearly identifiable

Flight was initiated by 103 workers, comprising 71% of *control* (*n* = 47 of 66) and 76% of *pesticide* workers (*n* = 56 of 74), revealing a similar propensity to fly between treatments (GLM: *z*_137_ = 0.5, *p* = 0.62; Supporting information Table S2). Body size was a significant predictor of propensity to fly, with the likelihood of flying increasing with *ITS* (GLM: *z*_137_ = 2.2, *p* = 0.03; Supporting information Table S2). This translated to an estimated probability of *control* workers initiating flight of 0.49, 0.77, and 0.92 for workers with a 4 mm, 5 mm, and 6 mm ITS, respectively, with a similar pattern observed for *pesticide* workers (Figure 3). We found that 43% (*n* = 24 of 56) of *pesticide* workers terminated the flight test within the 100‐m threshold compared with just 26% (*n* = 12 of 47) of *control* workers, representing a significantly higher flight termination by *pesticide* compared with *control* workers within the first 100‐m distance of the test (GLM: *z*_100_=−2.1, *p* = 0.04; Supporting information Table S2). For instance, a *control* worker with 5 mm *ITS* had an estimated probability of 0.81 of flying >100 m, compared with just 0.62 for a *pesticide* worker of the same *ITS*. We further found that larger *ITS* significantly increased the probability of flying >100 m (GLM: *z*_100_ = 2.3, *p* = 0.02), with no clear significant difference in this relationship between treatments (GLM: treatment*ITS: *z*_99_ = 1.9, *p* = 0.06).

#### 3.2.3 Flight endurance & velocity

Inspection of the 67 bees that flew >100 m showed an uneven *ITS* distribution between treatments, with a significant bias of larger *pesticide* workers (mean *ITS* of 4.83 ± 0.05 mm vs. 4.99 ± 0.04 mm for *control* vs. *pesticide* workers, respectively; LM: *t*_65_ = 2.4, *p* = 0.02). We therefore took a conservative approach and ran two separate analyses on: (a) the full dataset including all 67 bees (*control* = 35, *pesticide* = 32); and (b) a subset of the data (*control* = 26, *pesticide* = 27) in which we attempted to normalize the worker *ITS* distribution by removing the smallest 10% (*n* = 6 *control & 1 pesticide*) and largest 10% (*n* = 3 *control* & 4 *pesticide*) of workers; resulting in no significant difference in worker *ITS* between treatments (4.86 ± 0.03 mm vs. 4.94 ± 0.03 mm for *control* vs. *pesticide* workers, respectively; LM: *t*_51_ = 1.8, *p* = 0.08; Table 1). Normalizing the dataset allowed us to better meet the assumptions of our linear models; therefore, here we present the analysis using the data subset and provide the results using the full dataset in Supporting information Figure S3 and Table S1, which showed the same directional pattern in flight performance between treatments.

**Figure 4:**
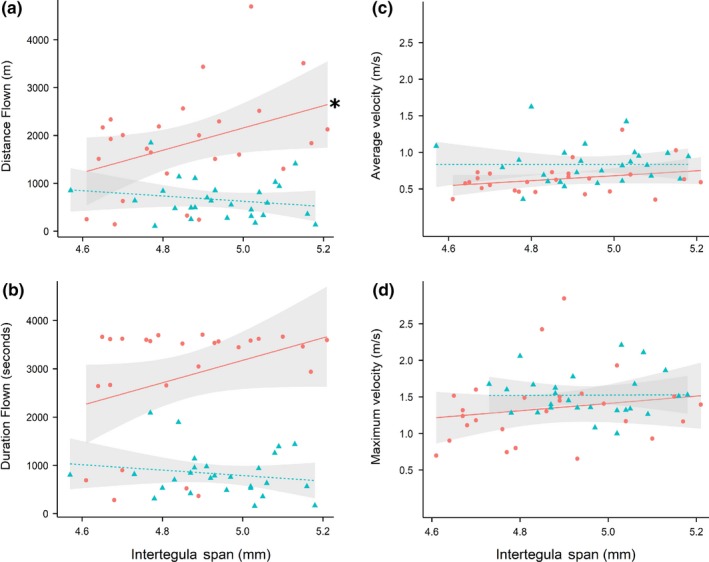
Scatterplot showing key flight performance indicators of endurance (distance flown in meters (A); duration flown in seconds (B)) and average and maximum velocity in meters per second (C‐D) against worker body size (*intertegula span*) for both the *control* (red circle) and *pesticide*‐treated (blue triangle) groups. Data plotted are for the subset of bees with normalized *ITS* between treatments (number of workers = 26 *control*; 27 *pesticide*), and linear fitted lines (*control* = solid red, *pesticide* = dashed blue) with associated standard error (shaded bands) are the estimates of linear models. An asterisk (*) indicates a significant (*p* < 0.05) relationship

*Pesticide* workers flew a significantly lower mean (±*SEM*) total distance at just 659.1 ± 78.7 m compared with 1,833.9 ± 207.6 m for *control* (LMM: *t*_48_ = −5.6, *p* < 0.001; Figure 4a; Supporting information Table S1). The effect of pesticide exposure on distance flown was mirrored in the effect on duration flown, with a mean (±*SEM*) flight duration of just 822.0 ± 90.8 s for *pesticide*‐exposed workers being considerably shorter than 2,852.2 ± 234.4 s for *control* workers (Figure 4b; Supporting information Table S1). Durations flown across all workers (Figure 4b) showed a striking difference between treatments, with a proportion of just 0.04 of *pesticide* workers flying > 2000 s, while 0.81 of *control* workers surpassed this duration (GLMM: *z*_49_ = −4.0, *p* < 0.001; Supporting information Table S1). Furthermore, a proportion of 0.65 of *control* workers flew for the full 60 min permitted, whereas critically not one *pesticide‐*exposed worker achieved this.

Interestingly, the effect of worker body size on distance flown appeared to differ between *pesticide* and *control* groups, as indicated by a significant treatment*ITS interaction (LMM: *t*_47_ = −2.2, *p* = 0.03; Figure 4a; Supporting information Table S1). Separate analysis of each treatment group showed that while increasing *ITS* resulted in significantly higher total distances for *control* workers (LMM: *t*_22_ = 2.2, *p* = 0.04), this relationship was not found for *pesticide*‐exposed workers (LMM: *t*_23_ = −1.0, *p* = 0.31; Figure 4a). The effect of *ITS* on total duration flown showed the same general trend as that found for distance (Figure 4b; Supporting information Table S1); however, the difference in effect between treatments was less strong (GLMM: *z*_48_ = −1.7, *p* = 0.09). Separate analyses for each treatment group found no significant relationship between *ITS* and the proportion of bees flying >2,000 s for both treatments (GLMM: *control*: *t*_23_ = 1.5, *p* = 0.13; *pesticide*: *t*_24_ = −1.1, *p* = 0.26).

**Figure 5:**
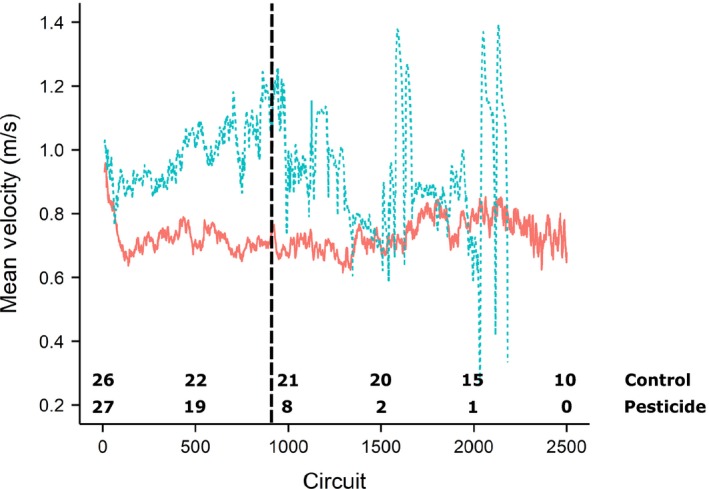
Mean velocity (m/s) flown by each treatment group (*control* = solid red, *pesticide* = dashed blue) plotted for each consecutive circuit for just the first 2,500 circuits. Numbers at the bottom of the graph refer to the number of bees still flying on the corresponding circuit, and the data plotted are for the subset of bees with normalized *ITS* between treatments (starting number of workers = 26 *control*; 27 *pesticide*). Vertical line represents the first 900 circuits used in the analysis for initial individual velocity, and the associated error per mean circuit velocity is not shown

When considering the velocity of individuals across the total flight period, we found *pesticide*‐exposed workers attained a significantly higher mean (± s.e.m) velocity of 0.84 ± 0.05 m/s per worker compared with 0.63 ± 0.04 m/s for *controls* (LMM: *t*_48_ = 3.0, *p* = 0.005; Figure 4c; Supporting information Table S1). For maximum velocity, we found no significant difference between treatments (LMM: *t*_47_ = 1.6, *p* = 0.12; Figure 4d; Supporting information Table S1). However, it was intriguing that the average maximum velocity for *pesticide*‐exposed workers was higher than *controls* (mean ± *SEM* = 1.52 ± 0.06 m/s vs. 1.34 ± 0.09 m/s). This observation is consistent with our previous analysis looking at mean velocity over the total flight period and motivated us to examine at what stages in flight these differences in velocity may occur. *Pesticide* workers appeared to maintain a higher velocity compared with *controls* during the initial phase (the earlier circuits) of the flight test (see Figure 5). *Pesticide* workers also showed a sharp decline in velocity around 900 circuits (760 m) as a large proportion terminated flight. Therefore, focusing on the first 900 circuits, we reveal that *pesticide* workers did fly significantly faster compared with *control* workers (LMM: *t*_753_ = 3.5, *p* = 0.001; Supporting information Table S3), with this difference between treatments maintained over these circuits (treatment*circuit interaction: *t*_752_ = 1.9, *p* = 0.07). Neither mean nor maximum velocity was significantly predicted by worker *ITS* (LMM: *t*_48_ = 1.6, *p* = 0.12 & *t*_47_ = 1.0, *p* = 0.32, respectively; Figure 4c, d; Supporting information Table S1), and there appeared to be no effect of *ITS* on velocity over the first 900 circuits (LMM: *t*_753_ = 0.5, *p* = 0.62; Supporting information Table S3).

## 4 DISCUSSION

Despite the importance of bumblebee foraging ability in providing a key pollination service (Garibaldi et al., 2013; Kleczkowski, Ellis, Hanley, & Goulson, 2017; Stanley et al., 2015), this study, to our knowledge, is the first to test how a specific stressor directly affects the properties of flight in bumblebees. Our findings demonstrate that acute exposure to the neonicotinoid pesticide, imidacloprid, was sufficient to significantly impact overall flight endurance, reducing flight distance and duration to around a third of what *control* workers were able to achieve. While both *control* and *pesticide*‐exposed workers were equally motivated to fly initially, *pesticide*‐exposed workers had a higher probability of terminating flight before the end of the 60‐min flight test, which was even evident within the first 100 m. Intriguingly, *pesticide* workers exhibited a higher mean velocity compared with *control* workers, which was underpinned by faster flight speeds over the course of the first ¾ km, both during and after which we observed a considerable proportion of pesticide workers terminating their flight. Furthermore, our results suggest that pesticide exposure may negate the capability of larger workers to fly longer distances than their smaller sister workers.

The degree of impact that an acute neonicotinoid exposure had on reducing bumblebee worker flight endurance observed in our study did come as a surprise, as a previous honeybee study showed acute exposure to thiamethoxam increased flight endurance (Tosi et al., 2017). One possible explanation for these contrasting results is the structural differences between thiamethoxam and imidacloprid compounds, which bind to different sites on nicotinic acetylcholine receptors (nAChRs) with variable affinity (Iwasa, Motoyama, Ambrose, & Roe, 2004; Kayser et al., 2004; Marletto, Patetta, & Manino, 2003; Wiesner & Kayser, 2000). Indeed, studies have previously shown bumblebees to be less sensitive to thiamethoxam compared to imidacloprid when considering effects on brood production and food consumption (Heard et al., 2017; Laycock, Cotterell, O'Shea‐Wheller, & Cresswell, 2014), and have found contrasting dose‐dependent responses when considering effects on bumblebee nonflight thermogenesis (Potts et al., 2018). That said, the only other flight mill study to test neonicotinoid effects on honeybee flight capacity also used imidacloprid (as in our study), yet found no effect on workers free from infection with *Varroa destructor* (Blanken et al., 2015). Therefore, this reinforces the view that responses to pesticide exposure can vary considerably even between closely related genera. Indeed, both laboratory (Cresswell et al., 2012) and field (Rundlöf et al., 2015) studies have highlighted differences in neonicotinoid effects between honeybees and bumblebees, with large interspecific differences in the toxicity of pesticides over time (Heard et al., 2017). Together this emphasizes the growing appreciation that reported effects on honeybees cannot always be extrapolated to other wild bees and highlights the danger of using honeybees as lone indicator species for insect pollinator responses to pesticides (Gill et al., 2016; Heard et al., 2017; Raine & Gill, 2015).

Our flight tests suggest that imidacloprid‐exposed bumblebee workers experienced a rapid demotivation to fly as the test progressed and/or tired quickly leading to premature physical exhaustion. Our study was not designed specifically to test these two non‐mutually exclusive explanations; however, given that only 4% of pesticide‐exposed workers flew >2,000 s (control = 81%) and that not one individual completed the 60 min test (control = 65%), our findings suggest that physical ability may have been affected, which could then have subsequently led to demotivation. We found no difference in initial motivation to fly and in fact pesticide‐exposed workers flew faster than control workers, implying that immediate motor function was not impaired per se, but instead that imidacloprid reduced flight stamina. Neonicotinoids have been implicated in affecting honeybee energy metabolism (Derecka et al., 2013), and imidacloprid has been shown to reduce mitochondrial activity, impairing respiratory processes, and causing rapid mitochondrial depolarization in neurons of bumblebees and honeybees (Moffat et al., 2015; Nicodemo et al., 2014). Given the high energy expenditure required during flight, a reduction in mitochondrial functioning and the consequent inhibition of ATP production in flight muscles could lead to rapid muscle exhaustion, which might explain our findings of significantly reduced endurance. Additionally, neonicotinoids can impair nonflight thermogenesis in bees (Tosi et al., 2016), with bumblebee exposure to imidacloprid at a concentration of just 5 ppb resulting in a drop in thoracic temperature by around 1.5–2°C, equating to a subsequent 15%–20% reduction in metabolic rate (Potts et al., 2018). This impact on bumblebee thermogenesis and metabolism may consequently compromise the performance of thoracic flight muscles, which must be raised to and maintained at ~35°C for successful flight (Heinrich, 1975; Potts et al., 2018). Imidacloprid can also induce the down‐regulation of genes involved in sugar metabolizing pathways in honeybee larvae (Derecka et al., 2013), which if true for bee adults could seriously impact flight performance that requires muscles to function at high glycolytic rates (Staples & Suarez, 1997). It is interesting to note that buzz pollination by bumblebees, whereby the creation of resonant vibrations from the flight muscles dislodges pollen from anthers (Morgan, Whitehorn, Lye, & Vallejo‐Marín, 2016), has also been reported to be impaired by neonicotinoid exposure (Whitehorn, Wallace, & Vallejo‐Marin, 2017).

Neonicotinoids are the agonists of insect nicotinic acetylcholine receptors (Déglise, Grünewald, & Gauthier, 2002) and can acutely increase neuronal activity (Matsuda et al., 2001; Moffat et al., 2016). A resultant effect of this may be individual hyperactivity of specific tasks, which could explain our observations of higher velocity in exposed workers during the initial phase of the flight test, and has been previously suggested to underpin neonicotinoid effects on honeybee flight and locomotor activity (Lambin, Armengaud, Raymond, & Gauthier, 2001; Suchail, Guez, & Belzunces, 2001; Tosi et al., 2017). Bumblebee colony level exposure to imidacloprid also leads to a higher number of workers going out to forage (Gill et al., 2012), a pattern that could be an adaptive response to filling a foraging deficit, but could also be maladaptive hyperactive behavior. Interestingly, Crall et al. (2018) found that while chronic exposure to imidacloprid increased movement speed in nest bumblebee workers, it actually decreased the amount of time workers were active, showing similar patterns of effects to our own findings. Similarly, when investigating neonicotinoid effects on nonflight thermogenesis in bumblebees, Potts et al. (2018) reported that the dose–response relationship for dietary imidacloprid exposure exhibited a biphasic hormesis, whereby low‐dose stimulated warming rates and a high dose led to inhibition. Heat generation in bumblebees is attained through contraction of thoracic flight muscles. Hence, the dose–response relationship demonstrated by Potts et al. (2018) may corroborate our findings of stimulatory behavior at the start of the experiment followed by the inhibition of flight as the trials progressed, with the neonicotinoid active ingredient accumulating at the target site during the course of the trial. Alternatively, our study may suggest a potential cost to hyperactivity, as exposed workers terminated flight prematurely, which may have been due to increased energy expenditure during the initial phase leading to faster muscle fatigue and energy depletion, but further testing would be needed to understand this. In sum, our results highlight the importance of looking at the pattern of flight dynamics, rather than experimental end points, to better understand the mechanisms behind how neurotoxic insecticides act and their temporal sublethal effects (Suchail et al., 2001; Wen & Scott, 1997).

Bumblebees reportedly exhibit a certain degree of alloethism, whereby worker body size can determine divisions in colony tasks (Goulson et al., 2002; Herrmann, Haddad, & Levey, 2018; Peat, Tucker, & Goulson, 2005). Larger workers of a colony are considered more likely to become committed foragers (Jandt & Dornhaus, 2009; Spaethe & Weidenmuller, 2002), and there have been reports of foraging rate, distance, and efficiency (nectar collected per unit time) increasing with body size (Goulson et al., 2002; Greenleaf et al., 2007; Jandt & Dornhaus, 2009; Kapustjanskij, Streinzer, Paulus, & Spaethe, 2007; Spaethe & Weidenmuller, 2002; Worden, Skemp, & Papaj, 2005). While our study found no clear relationship with flight velocity and body size, we did find that both the propensity to fly and total flight distance were positively related in *control* workers, which might provide a mechanistic explanation as to why foragers tend to be the larger colony workers. Critically, however, we found no such significant relationships in *pesticide*‐exposed workers, suggesting that the negative effect of neonicotinoid exposure on flight actually increased in magnitude as workers increased in body size. Intriguingly, a previous study showed that neonicotinoid‐induced impairment to spatial learning behavior in bumblebees appeared to be exhibited more highly in the largest colony workers (Samuelson, Chen‐Wishart, Gill, & Leadbeater, 2016). Together these findings raise the question as to whether larger bumblebees are more susceptible to pesticide effects. With pesticide exposure seemingly counteracting the increased flight performance with body size, the production of larger bees could be seen as wasted energetic investment for the colony. Further investigation is required to look at this; however, as while the interactive effect of pesticide and body size was detected in the subset of workers analyzed, this effect seemed to be lost when considering the full dataset, a discrepancy that may stem from biases in worker size between treatments as a consequence of the flight trial filtering process.

Bumblebee foraging ranges are difficult to accurately measure, and further knowledge of this important behavior is critical for predicting colony success and pollination services in changing landscapes. Our flight mill setup showed *control* workers to fly a mean total distance of 1.8 km, which appears to sensibly conform to other estimates of bumblebee foraging ranges. Estimated foraging ranges using different techniques including harmonic radar (Osborne et al., 1999), mark–recapture (Kreyer, Oed, Walther‐Hellwig, & Frankl, 2004; Osborne et al., 2008), and use of microsatellite genetic markers (Darvill, Knight, & Goulson, 2004) for *Bombus terrestris* vary from 0.34 to 2.2 km. As bumblebees are central place foragers, foraging trips require not only reaching a resource, but also returning to the nest site after collection of food or other resources. The minimum round‐trip flight distances associated with the above foraging ranges could therefore span from around 0.68 to 4.4 km. Given that our measures for control workers fall in the middle of these estimates, we are confident that our flight mill test setup can provide us with meaningful insights into the effects of stress on flight capabilities that can occur in the field. Pesticide‐exposed workers flew less distance than the lower limit of this estimated foraging range, with imidacloprid exposure reducing total flight distance by nearly 1.2 km on average. This corresponds to a 64% reduction in comparison with the *control,* which would lead to a notable 87% decline in the total foraging area accessible to a colony (using the colony as the epicenter). Pesticide exposure will therefore place increased stress on bumblebee colonies, with foragers potentially being unable to reach resources they previously could, or unable to return to the nest following exposure feeding on contaminated flowers. Not only would this reduce the abundance, diversity, and nutritional quality of food available to a colony, but could also reduce the pollination service the colony is able to provide (Blanken et al., 2015; Tosi et al., 2017; van der Sluijs et al. 2013). Looking at the effects of chronic exposure would provide further insights, as bees in the wild would likely be exposed to treated or contaminated flowering plants throughout the season (Simon‐Delso et al., 2015; Stanley, Gunning, & Stout, 2013; Tison et al., 2016). Furthermore, workers can be exposed to multiple pesticides concurrently when foraging (Botías et al., 2017; David et al., 2016; Hladik et al., 2016), yet an often‐overlooked issue is that of possible interactive effects between chemicals (Iwasa et al., 2004; Sgolastra et al., 2017), particularly when considering sublethal end point (Gill & Raine, 2014; Gill et al., 2012). An important next step, therefore, is to investigate interactive effects of commonly used pesticide classes on the dynamics of bee flight.

## CONFLICT OF INTEREST

No competing interests declared.

## AUTHOR CONTRIBUTIONS

RJG conceived the project; DK analyzed the data; HC, IP, ARR, and SG developed the experimental setup; HC performed the experiment; DK, HC, and RJG wrote the manuscript.

## EXPERIMENTAL ANIMALS

All procedures involving experimental animals were performed in compliance with local animal welfare laws, guidelines, and policies.

## Data Availability

Flight mill data files: Dryad https://doi.org/10.5061/dryad.gd702q0.
