# Supplementary material for: Pesticide exposure affects flight dynamics and reduces flight endurance in bumblebees
Source: Ecol Evol. 2019 Apr 29;9(10):5637–50. doi: 10.1002/ece3.5143 (PMC6540668; doi:10.1002/ece3.5143)
Supplement: Supplementary file 1 [file ECE3-9-5637-s001.pdf]

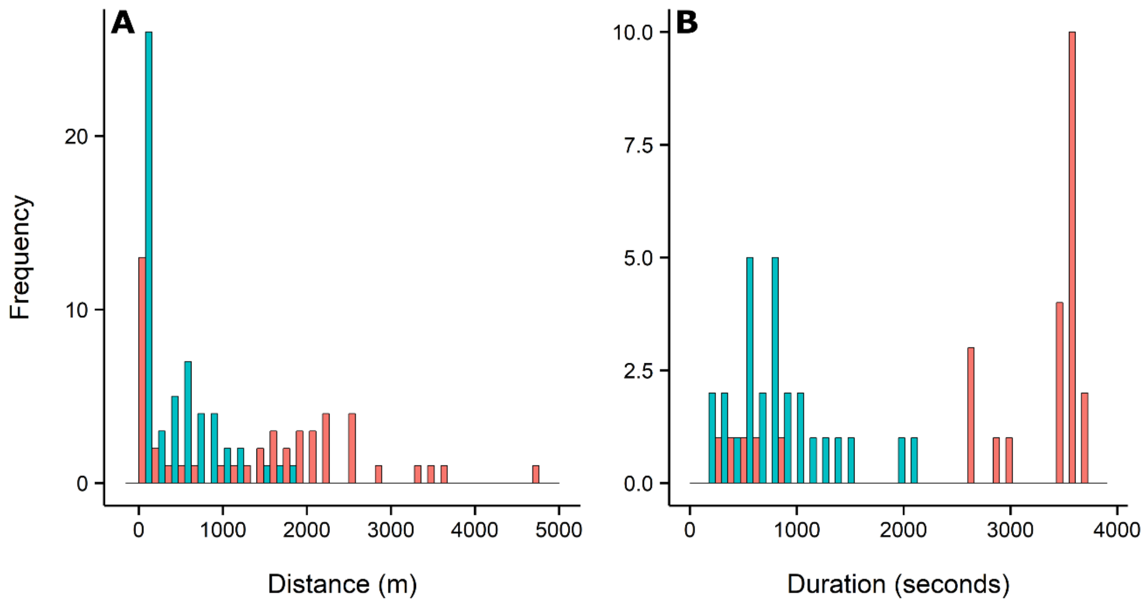

Figure S1. Frequency distributions of distance (A) and duration (B) flown by workers during the flight trials, considering both *control* (red) and *pesticide* exposed (blue) workers. Distributions highlight a spike in the number of workers that terminated flight within the first 100m (A), and demonstrate a bimodal distribution when considering total duration flown (B). For distance flown, all workers that initiated flight were considered (n=103, Table 1 – filter step 5), consisting of 47 *control* and 56 *pesticide* treated workers. For duration flown, data shown is for the subset of bees we used (n=53, Table 1 – filter step 7), consisting of 26 *control* and 27 *pesticide* treated bees, but the same distribution was observed when considering the full dataset (n=67, Table 1 – filter step 6).
