# Supplementary material for: Pesticide exposure affects flight dynamics and reduces flight endurance in bumblebees
Source: Ecol Evol. 2019 Apr 29;9(10):5637–50. doi: 10.1002/ece3.5143 (PMC6540668; doi:10.1002/ece3.5143)
Supplement: Supplementary file 2 [file ECE3-9-5637-s002.pdf]

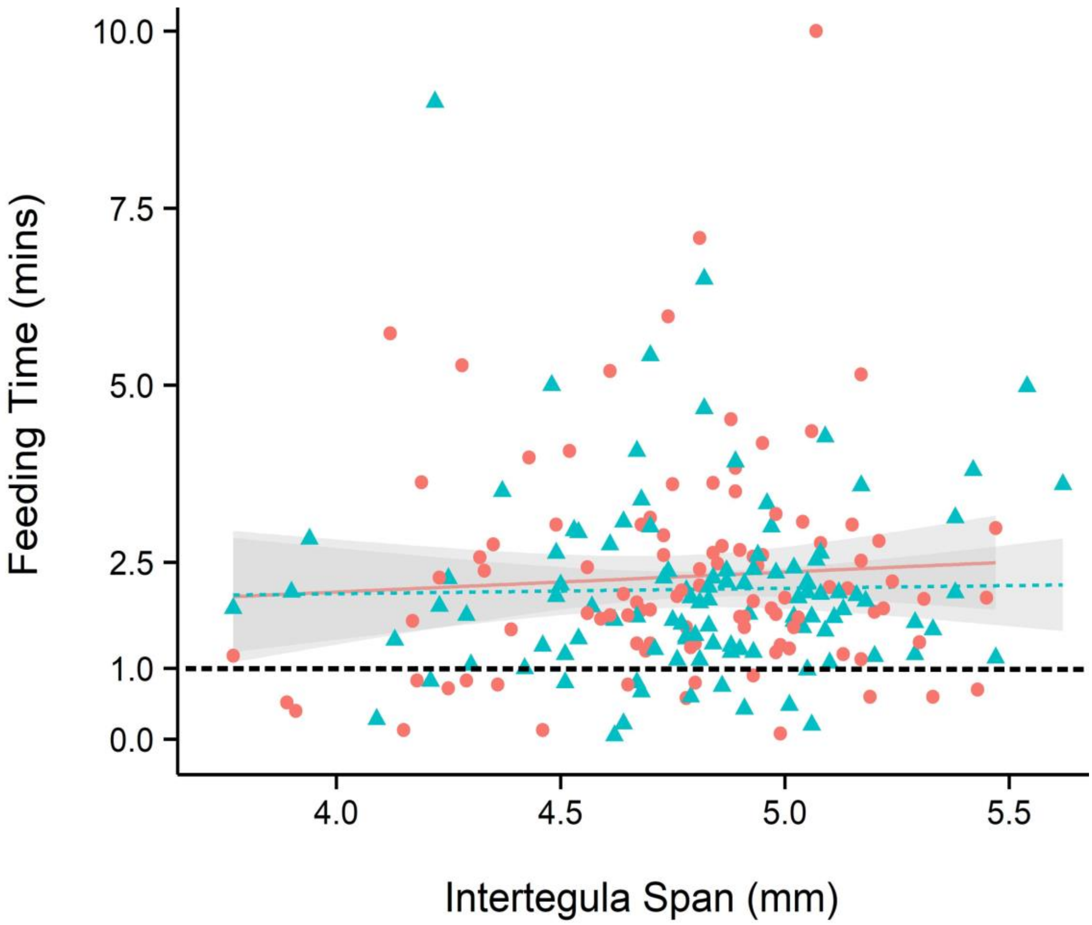

Figure S2. Scatter plot showing no significant relationship between length of feeding time and body size (*ITS*) for *control* (red circle) and *pesticide* exposed (blue triangle) workers. There were 222 bees selected for testing (*control* = 111, *pesticide* = 111), of which 209 initiated feeding (*control* = 102, *pesticide* = 107). The dashed black line corresponds with a feeding time of 60 seconds, and all bees that fed below this (*control* =16, *pesticide* = 13) were not considered in the main flight analysis. Linear fitted lines (*control* = solid red, *pesticide* = dashed blue) with associated standard error (shaded bands) are estimates of linear models.
