# Supplementary material for: Pesticide exposure affects flight dynamics and reduces flight endurance in bumblebees
Source: Ecol Evol. 2019 Apr 29;9(10):5637–50. doi: 10.1002/ece3.5143 (PMC6540668; doi:10.1002/ece3.5143)
Supplement: Supplementary file 3 [file ECE3-9-5637-s003.pdf]

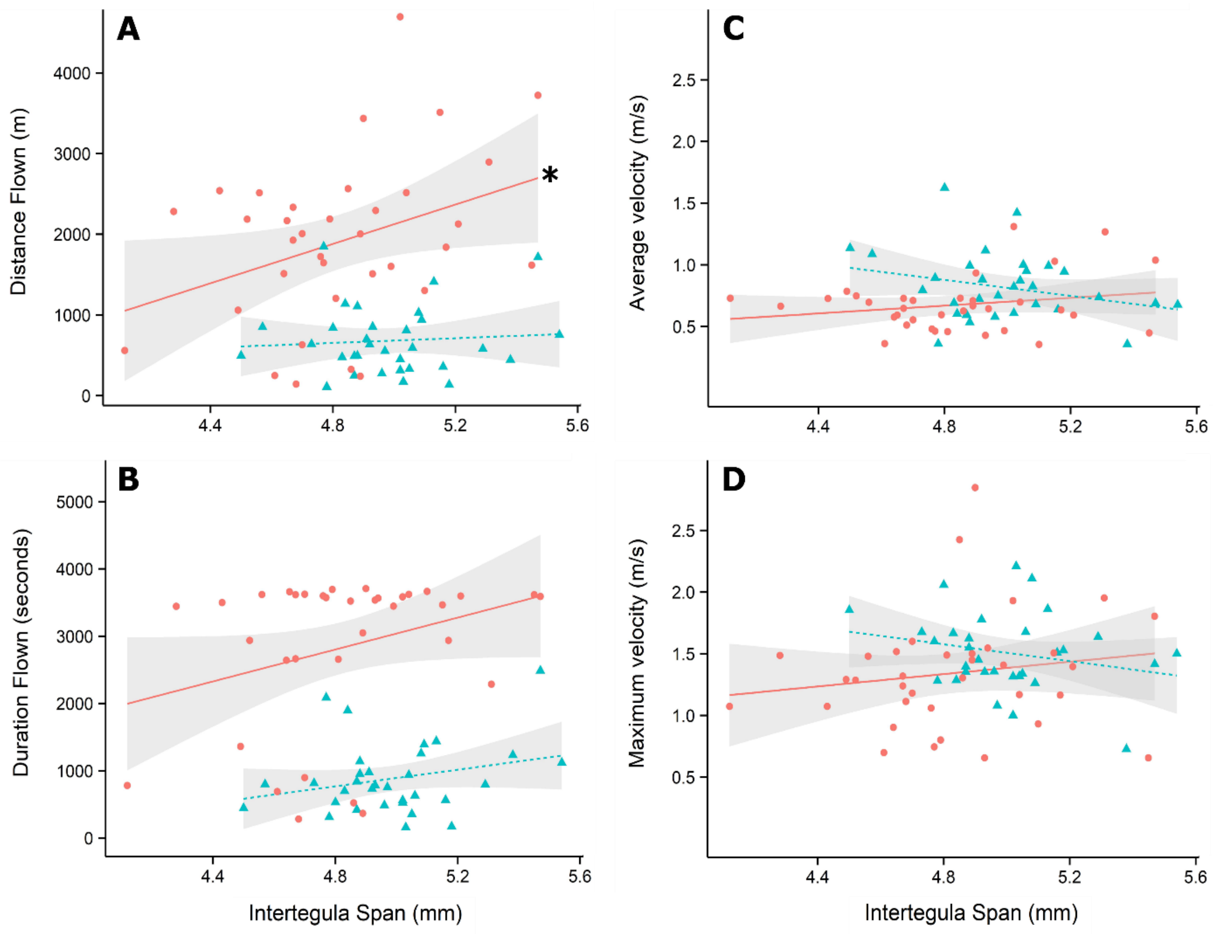

Figure S3. Scatterplot showing key flight performance indicators of endurance (distance flown in meters (A); duration flown in seconds (B)) and average and maximum velocity in meters per second (C-D) against worker body size (*ITS*) for both the *control* (red circle) and *pesticide* treated (blue treatment) groups. Data plotted are for the full dataset of bees (n = 35 *control*; 32 *pesticide*; see Table 1 – filter step 6), and linear fitted lines (*control* = solid red, *pesticide* = dashed blue) with associated standard error (shaded bands) are estimates of linear models. An asterisk (*) indicates a significant ($p<0.05$) relationship.
