# Supplementary material for: Pesticide exposure affects flight dynamics and reduces flight endurance in bumblebees
Source: Ecol Evol. 2019 Apr 29;9(10):5637–50. doi: 10.1002/ece3.5143 (PMC6540668; doi:10.1002/ece3.5143)
Supplement: Supplementary file 4 [file ECE3-9-5637-s004.docx]

**Table S1. Linear mixed model* summary output for the effect of neonicotinoid (*pesticide*) exposure, worker body size (*ITS*) and the interaction between these two variables on key flight performance metrics.**

|  | | ***Distance Flown*** | | | ***Duration Flown*** | | | ***Average Velocity*** | | | ***Maximum velocity*** | | |
| --- | --- | --- | --- | --- | --- | --- | --- | --- | --- | --- | --- | --- | --- |
|  |  | *Estimate* | *t value* | *P value* | *Estimate* | *z value* | *P value* | *Estimate* | *t value* | *P value* | *Estimate* | *t value* | *P value* |
| ***Additional Dataset***  ***(n=72)*** | *Intercept* | -8.347 | -0.393 | 0.695 | -14.209 | -2.044 | 0.041 | 0.792 | 2.968 | 0.004 | 0.947 | 2.774 | 0.007 |
|  | *Treatment - Pesticide* | -17.876 | -7.179 | ***<0.001*** | -4.969 | -4.729 | ***<0.001*** | 0.078 | 2.483 | ***0.015*** | 0.062 | 1.548 | 0.126 |
|  | *ITS* | 10.250 | 2.344 | ***0.022*** | 3.250 | 2.208 | ***0.027*** | 0.003 | 0.051 | 0.959 | 0.040 | 0.568 | 0.572 |
|  | *Treatment * ITS* | -7.288 | -0.829 | 0.410 | -0.569 | -0.168 | 0.866 | -0.134 | -1.223 | 0.226 | -0.048 | -0.334 | 0.739 |
| ***Full Dataset***  ***(n=67)*** | *Intercept* | -8.14 | -0.347 | 0.730 | -14.18 | -1.951 | 0.051 | 0.860 | 2.936 | 0.005 | 1.060 | 4.511 | <0.001 |
|  | *Treatment - Pesticide* | -18.50 | -6.903 | ***<0.001*** | -5.10 | -4.609 | ***<0.001*** | 0.077 | 2.296 | ***0.025*** | 0.050 | 1.855 | 0.068 |
|  | *ITS* | 10.337 | 2.135 | ***0.036*** | 3.27 | 2.120 | ***0.034*** | -0.010 | -0.166 | 0.868 | 0.006 | 0.124 | 0.902 |
|  | *Treatment * ITS* | -12.45 | -1.236 | 0.221 | -0.91 | -0.255 | 0.799 | -0.226 | -1.813 | 0.074 | -0.149 | -1.448 | 0.152 |
| ***Subset***  ***(n=53)*** | *Intercept* | -23.85 | -0.522 | 0.604 | -13.69 | -0.938 | 0.348 | -0.065 | -0.124 | 0.902 | 0.419 | 0.578 | 0.566 |
|  | *Treatment - Pesticide* | -17.19 | -5.618 | ***<0.001*** | -5.11 | -4.016 | ***<0.001*** | 0.103 | 2.954 | ***0.005*** | 0.074 | 1.580 | 0.120 |
|  | *ITS* | 13.26 | 1.412 | 0.164 | 3.13 | 1.031 | 0.303 | 0.174 | 1.603 | 0.115 | 0.148 | 0.997 | 0.324 |
|  | *Treatment * ITS* | -40.96 | -2.242 | ***0.029*** | -14.16 | -1.720 | 0.085 | -0.166 | -0.764 | 0.448 | -0.227 | -0.722 | 0.474 |

**Note:** In all cases, bees were only considered in analyses if they had an ‘ideal’ tag fitting (tag rating 1) and flew > 100m. The top row of results (Additional Dataset) considered all bees that fed, regardless of how long they fed for (*control* = 37, *pesticide* = 35). The middle row of results (Full Dataset) considered all bees that fed > 60 seconds (*control* = 35, *pesticide* = 32). The bottom row of results (Subset) considered all bees that fed > 60 seconds with the smallest 10% and largest 10% of workers removed (*control* = 26, *pesticide* = 27). *****GLMM was used for duration flown under a binomial family distribution
