# Supplementary material for: Pesticide exposure affects flight dynamics and reduces flight endurance in bumblebees
Source: Ecol Evol. 2019 Apr 29;9(10):5637–50. doi: 10.1002/ece3.5143 (PMC6540668; doi:10.1002/ece3.5143)
Supplement: Supplementary file 5 [file ECE3-9-5637-s005.docx]

**Table S2. Generalised linear model summary output for the effect of neonicotinoid (*pesticide*) exposure, worker body size (*ITS*) and the interaction between these two variables on the propensity of individuals to initiate flight and to fly >100m threshold distance.**

|  |  | *Estimate* | *Std.Error* | *z value* | *P value* |
| --- | --- | --- | --- | --- | --- |
| ***Propensity to Fly***  ***(n=140)*** | *Intercept*  *Treatment - Pesticide*  *ITS*  *Treatment * ITS* | -4.981  0.194  1.233  0.930 | 2.723  0.391  0.570  1.151 | -1.829  0.496  2.163  0.808 | 0.067  0.620  ***0.031***  0.419 |
| ***Flight >100m***  ***(n=103)*** | *Intercept*  *Treatment - Pesticide*  *ITS*  *Treatment * ITS* | -7.620  -0.959  1.816  3.169 | 3.738  0.454  0.784  1.703 | -2.038  -2.115  2.318  1.862 | 0.042  ***0.035***  ***0.020***  0.063 |
