# Supplementary material for: Pesticide exposure affects flight dynamics and reduces flight endurance in bumblebees
Source: Ecol Evol. 2019 Apr 29;9(10):5637–50. doi: 10.1002/ece3.5143 (PMC6540668; doi:10.1002/ece3.5143)
Supplement: Supplementary file 6 [file ECE3-9-5637-s006.docx]

**Table S3. Linear mixed model summary output for flight velocity over time: initial velocity over the first 900 circuits was significantly affected by treatment, with *pesticide* treated bees flying faster over this period than *control* bees.**

|  | **Full model (value for all 900 circuits)** | | **Final model (average velocity taken for bands of 50 circuits)** | |
| --- | --- | --- | --- | --- |
|  | T-value | p-value | T-value | p-value |
| *Treatment - pesticide* | 2.803 | ***0.007*** | 3.459 | ***0.001*** |
| *Circuit* | -0.581 | 0.564 | -0.328 | 0.745 |
| *ITS* | 0.085 | 0.933 | 0.502 | 0.618 |
| *Treatment * Circuit* | 1.735 | 0.089 | 1.862 | 0.072 |

**Note:** The model suffered from high eigenvalues and had trouble converging when considering all 900 repeated measures (Full model). Therefore, to enhance model fit and convergence we scaled the *circuit* variable and considered the average velocity of every 50^th^ circuit (i.e. each bee had a mean per circuit velocity for circuits 1 to 50, 51 to 100, 101 to 150 and so on) resulting in 18 repeated measures (Final model). This analysis considered the subset of workers (*control* = 26, *pesticide* = 27; see Table 1 – filter step 7).
