# Supplementary material for: Pesticide exposure affects flight dynamics and reduces flight endurance in bumblebees
Source: Ecol Evol. 2019 Apr 29;9(10):5637–50. doi: 10.1002/ece3.5143 (PMC6540668; doi:10.1002/ece3.5143)
Supplement: Supplementary file 7 [file ECE3-9-5637-s007.docx]

**Appendix 1**

**Pesticide exposure alters flight dynamics and reduces flight endurance in bumblebees**

Daniel Kenna*, Hazel Cooley*, Ilaria Pretelli, Ana Ramos Rodrigues, Steve D. Gill & Richard J. Gill

**Statistical Analysis**

In all cases, model residuals were plotted to confirm the data met the parametric assumptions of the tests used, and model fit was assessed and optimised where possible. Where appropriate, normality tests were used to reveal distributions of the data, and those which appeared non-normal were suitably transformed as outlined below:

*Feeding time*

The feeding time response variable was Box-Cox transformed (Venables and Ripley, 2002) to the optimal exponent.

*Effect of tag fitting on flight behaviour*

The effect of tag fitting on flight behaviour was examined separately for both treatments. The effect of tag fitting on total distance flown was analysed using a linear model, with the response variable being square root transformed when considering the *pesticide* treatment, but being left untransformed when considering the *control* treatment.

*Average and maximum velocity*

Prior to maximum velocity analysis, one outlier was identified in the *pesticide* treatment group with a maximum velocity of 14 m/s. This value was around 12 m/s higher than any other individual, and being considered a reaction to stimulation as opposed to normal flight behaviour this value was removed from further analysis. When considering the full dataset of 67 bees (Table 1 – filter step 6), the average velocity and maximum velocity values were square root and cube root transformed respectively. When considering the additional dataset of 72 bees (Table S1) and the subset of 53 bees (Table 1 – filter step 7), both the average velocity and maximum velocity values had to be square root transformed.

*Total distance flown*

For analysis of all datasets, the total flight distance response variable was square root transformed.

Reference:-

Venables, W. N., & Ripley, B. D. (2002). Modern applied statistics with S, 4th ed. New York, NY: Springer. ISBN 0‐387.
